# Hospital-based care for hallucinogens and risk of mania and bipolar disorder: A population-based cohort study

**DOI:** 10.1371/journal.pmed.1004805

**Published:** 2025-12-02

**Authors:** Daniel T. Myran, Rachael MacDonald-Spracklin, Michael Pugliese, Maya Gibb, Jess G. Fiedorowicz, Tyler S. Kaster, Marco Solmi

**Affiliations:** 1 Department of Family and Community Medicine, North York General Hospital, Toronto, Canada; 2 Department of Family Medicine, University of Ottawa, Ottawa, Ontario, Canada; 3 Bruyère Health Research Institute, Ottawa, Ontario, Canada; 4 Ottawa Hospital Research Institute, Ottawa, Ontario, Canada; 5 ICES uOttawa, Ottawa Hospital Research Institute, Ottawa, Ontario, Canada; 6 School of Epidemiology and Public Health, Faculty of Medicine, University of Ottawa, Ottawa, Canada; 7 Department of Psychiatry, University of Ottawa, Ontario, Canada; 8 Department of Mental Health, The Ottawa Hospital, Ontario, Canada; 9 Department of Psychiatry, Faculty of Medicine, University of Toronto, Toronto, Ontario, Canada; 10 Temerty Centre for Therapeutic Brain Intervention, Centre for Addiction and Mental Health, Toronto, Ontario, Canada; 11 Department of Child and Adolescent Psychiatry, Charité Universitätsmedizin, Berlin, Germany; University of Montreal Hospital Centre Research Centre: Centre de Recherche du Centre Hospitalier de l'Universite de Montreal, CANADA

## Abstract

**Background:**

Hallucinogen use for both recreational and medical purposes is rapidly increasing globally, raising concerns about potential adverse effects. This study examined the risk of incident mania or bipolar disorder (BD) diagnosis associated with having an emergency department (ED) visit or hospitalization involving hallucinogens.

**Methods and findings:**

We used a population-based cohort study of all individuals aged 14–65 years with no baseline history of BD and registered in the Ontario Health Insurance Plan in Ontario, Canada, between 2008–2022. Incident mania (primary outcome) and incident BD (secondary outcome) were compared between individuals with acute care (an ED visit or hospitalization) involving hallucinogens and the general population using overlap propensity score weighted Cox proportional hazard models. Models were adjusted for age, sex, rural residence, income quintile, recent documentation of homelessness, and healthcare encounters for mental health or other substance use in the past five years. The study included 9,311,844 individuals of which 7,285 (0.08%) had acute care involving hallucinogens. Within 3-years of acute care involving hallucinogens, 1.43% (n = 104) of individuals had an incident episode of mania requiring acute care compared to 0.06% (n = 41) of individuals in the age-sex matched general population, a 25-fold increase in risk. After weighting, acute care for hallucinogens was associated with a 6-fold (weighted Hazard Ratio [HR] 5.97, 95% CI 3.29, 10.82) increase in risk of incident mania relative to individuals without hallucinogen acute care who had otherwise similar demographic and mental health histories. Associated increases were also observed for risk of an incident diagnosis of BD (HR 3.75 95%CI 2.49, 5.65, absolute proportion 2.50% versus 0.11%). The main limitation of the study is the risk associated with the exposure examined in this study may not generalize to the majority of people who use hallucinogens who do not require acute care.

**Conclusions:**

These findings suggest the need for ongoing caution regarding hallucinogen use in individuals at risk of bipolar disorder. They also have potential implications for clinical practice, research, and public health policy, including substance regulation and targeted education for high-risk groups in the context of rising hallucinogen use.

## Author summary

**Why was this study done?:**

- Hallucinogen use is increasing in both clinical and naturalistic (e.g., recreational or non-medical) settings.
- There is limited information about adverse effects associated with hallucinogen use, in particular the risk of inducing mania and bipolar disorder (BD).

**What did the researchers do and find?:**

- We used the healthcare records for 9 million people aged 14–65 to compare the risk of developing mania or BD over time in the 7,285 individuals who required emergency or hospital care after hallucinogen use to comparators.
- Within 3 years, people requiring acute care involving hallucinogen use were 6 times more likely to have an episode of mania, and 4 times more likely to be diagnosed with BD compared to the general population.
- Individuals with acute care involving hallucinogens were also at higher risk of mania and BD compared to people who required care for reasons other than hallucinogens use.

**What do these findings mean?:**

- Patterns of hallucinogen use that result in ED or hospital care may increase the risk of developing mania or BD.
- The findings highlight the need for a better understanding of adverse effects of hallucinogens given rapid increase in their use.
- The main limitation of the study is that the findings may not apply to the majority of people who use hallucinogens who do not require medical attention, and the findings may be due to ED or hospital care involving hallucinogen use occurring in individuals at greater risk for mania or BD rather than a direct risk of hallucinogens.

## Abstract 3

Using a population-based cohort study, Daniel Myran and colleagues report that emergency or hospital care after hallucinogen use was associated with an increased risk of incident mania or bipolar disorder diagnosis.

## Introduction

Hallucinogens are substances that influence mood and perception. Hallucinogens are broadly classified into dissociative drugs, including ketamine and phencyclidine (PCP), and serotonergic hallucinogens (psychedelics), including psilocybin, lysergic acid diethylamide (LSD), dimethyltryptamine (DMT, the main psychoactive ingredient in ayahuasca), and methylenedioxymethamphetamine (MDMA, also known as Ecstasy), among others [1–4]. Over the past two decades, there has been increasing public interest and adult use of hallucinogens in North America. Available evidence from the United States (US) suggests that this trend is being driven in particular, by LSD and psilocybin, which began increasing in 2015, while MDMA and PCP use declined over time [1]. Emergency department (ED) visits involving hallucinogen use were similarly stable in the early 2010s but increased by 54% between 2016 and 2,022 in California [5] and 86.4% between 2013 and 2,021 in Ontario, Canada [6]. In 2023, an estimated 5.9%, 1.8%, and 0.7% of adults in Canada reported past-year use of psychedelics, MDMA, and dissociative drugs, respectively [7], while 8.9% of US adults aged 19–30 reported past-year hallucinogen use [4]. The rising use of hallucinogens has highlighted evidence gaps on potential adverse mental health effects [8]. One of the possible long-term safety concerns of hallucinogen use is the development of bipolar disorder (BD) or inducement of mania in individuals with existing BD [9–12]. This concern has led to caution when including individuals with a history of mania or bipolar I disorder (BD I) in current clinical trials evaluating the therapeutic effects of hallucinogens [9].

Critically, most of evidence on the association between hallucinogen use and mania and BD is limited to small cross-sectional studies and case reports with poor representativeness of the general population [9–12]. A review of case reports identified 6 patients who experienced hypomania or mania, following the use of hallucinogens, many of the cases had adverse events onset after a singular use of hallucinogens with prolonged effects lasting months [13]. Half of cases had used LSD, and half of cases had co-morbid cannabis use, the latter being known to worsen hypomania and mania [14]. Two cross-sectional studies found that self-reported hallucinogen use was either not associated with BD, or mania, or only found an association among those with genetic vulnerability [15,16]. A recently published longitudinal study of US adults found that self-reported psychedelic use during the two-month period between study entry and follow up found that psychedelic use was associated with worsening of mania symptoms [17]. Overall, the few available studies have been generally limited by small sample sizes, and examining measures of hallucinogen use that might have lower clinical relevance (e.g., lifetime use or any use in past two months) or cannot establish temporality. The lack of high-quality evidence on potential adverse effects of hallucinogens, including the risk of mania, has important potential public health and clinical implications given the rapid increase in adult hallucinogen use in North America over the past decade [1–4], along with evidence indicating a growing number of individuals requiring emergency care related to hallucinogen use [5,6].

In this study we used a contemporary population-based cohort in Ontario (Canada’s most populous province) to examine the association between acute care (an emergency department visit or hospitalization) involving hallucinogen use and an incident mania diagnosis in an acute care setting, or a diagnosis of BD in any setting. We hypothesized that incident mania and bipolar diagnoses would be greater among those with acute care involving hallucinogen use compared to all-cause acute care and the general population.

## Methods

### Ethics statements

The use of the data in this project was authorized under section 45 of Ontario’s Personal Health Information Protection Act (PHIPA) and does not require review by a Research Ethics Board.

### Study design and population

This retrospective cohort study was conducted between January 2008 and December 2022, and included all Ontario residents between the ages of 14 and 65 who were registered during the study period for the Ontario Health Insurance Plan (OHIP) – the province’s universal health system, which covers an estimated 97% of Ontario residents. The lower age range was selected based on when mania typically first presents, and the upper limit was set to avoid potential misclassification of other conditions (e.g., dementia or delirium) [18]. We excluded all individuals with a prior diagnosis of BD in the past 5-years or past acute care involving hallucinogens. We required individuals to have been continuously registered for OHIP in the 5 years before their study index date (the first hallucinogen-related ED visit or matched date for comparison) to be included in the study to ensure capture and exclusion of individuals with prevalent BD diagnoses. The use of the data in this project was authorized under section 45 of Ontario’s Personal Health Information Protection Act (PHIPA) and does not require review by a Research Ethics Board. We followed the *Reporting of Studies Conducted using Observational Routinely-Collected Data (RECORD)* reporting guideline in this study (S1 File **RECORD Checklist**).

### Regulatory context

Throughout our study period the possession, sale, and production of hallucinogens were illegal under the Controlled Drugs and Substance Act (CDSA) [19,20], except for a very small number of individuals whom were either patients in a clinical trial, received an exemption to the CDSA under section 56 prior to January 2022 or received authorization through the special access (SAP) program from the Federal Government [19,21]. Starting in January 2022, the final year of our study, healthcare practitioners were able to request access to hallucinogens through the SAP program for individuals who they deemed to have a life-threatening condition when conventional therapies were ineffective, unsuitable, or unavailable [22,23].

### Data sources

The data in this study for ED visits, hospitalizations, outpatient visits and covariates were obtained from six individual-level databases. These datasets were linked using unique encoded identifiers and analyzed at ICES (formerly the Institute for Clinical Evaluative Science). ICES is an independent, non-profit research institute whose legal status under Ontario’s health information privacy law allows it to collect and analyze healthcare and demographic data, without consent, for health system evaluation and improvement.

### Exposures

The primary exposure in this study was an ED visit or hospitalization involving hallucinogen use. We used the *Canadian Institute for Health Information*’s “Hospital Stays for Harm Caused by Substance Use” indicator to determine the definition of a visit involving hallucinogen use. We identified any ED visit or hospitalization including the *International Classification of Diseases 10th revision* (ICD-10) codes F16.X (mental and behavioural disorders due to use of hallucinogens which include acute intoxication [F16.0], harmful use [F16.1], withdrawal and dependence [F16.2, F16.3, F16.4], induced psychosis [F16.5, F16.7], and other and unspecified [F16.6, F16.8, and F16.9]). We also identified hallucinogen-involved visits when the two available ICD-10 codes for hallucinogen poisoning T40.8 (poisoning by or adverse effects of LSD) or T40.9 (poisoning by or adverse effects of unspecific hallucinogens) were listed as the main or contributing reason for the visit or hospitalization. Notably, with the exception of the LSD poisoning code, ICD-10 coding does not specify which type of hallucinogen was used.

In April 2018 Ontario mental health hospital beds migrated from ICD-9 to ICD-10 coding. We identified admissions to adult mental health hospital beds in Ontario pre 2018 involving hallucinogens when ICD-9 codes 304.50 (hallucinogen dependence) or 305.30 (hallucinogen abuse) were listed as the main or contributing reason for hospitalization.

### Comparison groups

We used three comparison groups. The first comparison group matched members of the general population with no acute care involving hallucinogens. See **Methods A in** S1 Text for details on how index dates were assigned. Our second comparison matched individuals with an incident all-cause acute care for any reason excluding visits involving hallucinogens. The final comparison group included individuals with incident acute care due to cannabis identified when an ICD-10 or Diagnostic and Statistical Manual of Mental Disorders (DSM) code for cannabis was the main or contributing reason for the visit (see **Methods B in** S1 Text for codes) [24]. Prior work has shown positive associations between cannabis acute care and cannabis use and mania and bipolar disorder [25,26]. We used acute care due to cannabis as an active comparator to acute care involving hallucinogens to see if observed associations differed.

### Outcomes

Our primary outcome was an incident mania diagnosis in an acute care setting (ED, general hospital bed, or mental health hospital bed). We defined a mania diagnosis as a) one hospitalization with an ICD-10 code for a manic episode or b) two ED visits within a 2-year period both with an ICD-10 code for a manic episode with the diagnosis occurring at the time of the second ED visit. ICD-10 codes F30.X used to identify a manic episode included, F30.1 (manic episode without psychosis), F30.2 (manic episode with psychosis), F30.8 (manic episode other), F30.9 (manic episode unspecified), F31.1 (BD, current episode manic without psychosis), F31.2 (BD, current episode manic with psychosis), F31.6 (BD, current episode mixed) and ICD-9 codes 296.0 (BD, single manic episode), 296.4 (more recent episode manic), and 296.6 (most recent episode manic). We excluded codes for hypomania from our primary outcome definition [(F30.0, hypomania), and (F31.0, BD, current episode hypomanic)].

As secondary outcomes, we examined 1) incident acute care for BD and 2) incident care for BD in either an acute care or outpatient setting. For our secondary outcome, including outpatient care, we required either one acute care visit or two outpatient visits with a diagnosis of BD occurring within 2 years of each other, with the outcome occurring at the time of the second outpatient visit, see **Methods A in** S1 Text for codes for BD and mania. Health administrative coding for BD has been previously validated as having good sensitivity and specificity [27]. Individuals with codes for mania or BD at index (i.e., a co-diagnosis of mania or BD during acute care event involving hallucinogens) were excluded.

### Covariates

At the time of index exposure, we recorded sociodemographic and health information for all individuals. Covariates included age, sex, rural residence, neighbourhood income quintile, and whether they had recent documentation (5-year lookback from index date) of homelessness in an ED visit or hospitalization [28]. Long-term resident of Canada status (since 1985) was also included as a covariate, with data obtained from the *Immigration Refugees and Citizenship Canada’s Permanent Resident Database*. Health information was based on care received in the past five years, including outpatient mental health visits (family physicians, or psychiatrist), ED visits, and hospitalizations for substance use and mental health [29,30]. See **Methods C in** S1 Text for covariate definition codes.

### Statistical analysis

We matched individuals with acute care involving hallucinogens to members of the general population in a up to 1:10 ratio using a greedy match algorithm on exact age, sex, and index date of the incident acute care. We compared the characteristics of individuals with an incident acute care involving hallucinogens and the general population using descriptive statistics and standardized mean differences (SMD) [31]. Characteristics were recorded at the time of the incident acute care event or the assigned index date for matched members of the general population. For rurality and neighbourhood income quintiles, we created an additional category to capture individuals with missing data.

We compared incident diagnosis of mania or BD between individuals with acute care involving hallucinogens and our three comparator groups using overlap propensity score weighting. Overlap weighting, estimates the average treatment effect in the overlap population (ATO), which is recommended for examining effects for an exposure that is less plausibly applied at the population-level [32]. In overlap weighting unexposed individuals who are most similar to the exposed contribute more to analysis and those who are less similar contribute less. This results in a cohort where individuals whose baseline characteristics are at equipoise for exposure (equally likely to have or not have acute care for hallucinogens) contribute more analysis and individuals who are more likely to be either always exposed or unexposed contributing less to analysis [33].

To generate propensity scores, we used the following covariates; age (restricted cubic splines at 5, 27.5, 50, 72.5, 95 percentiles), sex, income quintile, rurality, immigration, homelessness, past 5-year acute care for seven substance categories (alcohol, opioids, cocaine, amphetamines, cannabis, polysubstance, and other), past 5-year mental healthcare including outpatient family medicine or psychiatry, and acute care for five mental health conditions (mood disorder, anxiety, self-harm, schizophrenia, and other). We ran one set of models weighted only for age and sex and another set of models weighted for all covariates. We used cause-specific Cox proportional hazard models with robust sandwich variance estimate to generate hazard ratios with 95% confidence intervals [34]. We confirmed there was no violation of the proportional hazards assumption by examining time-varying hazards and log-negative log plots. We calculated hazard ratios at 3-years after the index event to balance potential time until and establishment of diagnosis and temporal plausibility to exposure, as done previously [6,35]. All analyses were conducted using SAS Enterprise Guide 8.3 (SAS Institute, Cary, NC).

### Secondary and sensitivity analyses

We compared the risk of incident mania between individuals with an incident acute care visit involving hallucinogens to two alternate cohort comparison groups: 1) individuals with an incident all-cause acute care visit (e.g., not involving hallucinogens) and 2) individuals with an acute care visit involving cannabis. All-cause acute care visits were matched in a up to 1:10 ratio on age, sex, type of acute care (ED, general hospital bed, mental health hospital bed, and date of acute care (within +/- 30-days). We included all individuals with an acute care visit involving cannabis, without matching on age and sex, as they were more comparable in age and sex composition.

We completed four sensitivity analyses. First, we excluded individuals with prior acute care for major depression in the past 5-years, as it is possible that an individual’s first bipolar disorder presentation might have been for depression or a depressive disorder. Second, we completed analyses stratified by age group and sex for the primary outcome mania between hallucinogen acute care and the general population.

In response to peer review suggestions, we completed two additional sensitivity analyses. First, we completed an analysis where we defined incident mania as a single ED visit rather than requiring two ED visits spaced 2 years apart in order to eliminate the possibility of immortal time bias. Second, we excluded all data from March 2020 to December 2022 to account for possible influences of COVID-19 on health service use.

## Results

During the study period, a total of 9,311,844 individuals were eligible for inclusion in analyses of which 7,285 (0.08%) had incident acute care involving hallucinogens. Our primary analysis comparing individuals with acute care involving hallucinogens to general population members matched on age, sex and index date included a total of 78,201 individuals with a median follow-up of 7 (interquartile range 3–11) years, see **Fig A in** S1 Text for cohort flow. The most common reasons for hallucinogen acute care were harmful use (n = 2,594; 35.6%), intoxication (n = 1,513; 20.8%), hallucinogen poisoning (n = 1,413; 19.4%), and dependence or withdrawal (n = 870; 11.9%). Individuals with hallucinogen acute care were more likely to live in low-income neighbourhoods, have documented homelessness during a prior acute care encounter, be long-standing residents of Canada, and have had prior outpatient mental healthcare or acute care for substance use or a mental disorder in the past five years compared to the general population. **Table 1** presents cohort characteristics and **Table A in** S1 Text shows characteristics before and after overlap propensity score weighting.

**Table 1:** Characteristics of individuals with an incident acute care involving hallucinogens and the general population.

	Hallucinogen acute care (n = 7,285)	Matched general population (n = 70,916)	Standardized mean difference
	No. (%)		
**Reason for Hallucinogen ED Visit or Hospitalization** ^**A**^			
Intoxication	1,513 (20.8)	N/A	
Harmful Use	2,594 (35.6)		
Dependence or Withdrawal	870 (11.9)		
Hallucinogen-Induced Psychosis	465 (6.4)		
Amnesia, Other, Unspecified	291 (4.0)		
Hallucinogen Poisoning	1,413 (19.4)		
**Designated Mental Health Bed Hospitalization** ^**B**^	583 (8.0)		
**Location of Acute Care**			
Emergency Department	6,228 (85.5)	N/A	
Acute Care Hospital Bed	474 (6.5)		
Designated Mental Health Hospital Bed	583 (8.0)		
**Sex**			
Male	5,206 (71.5)	50,716 (71.5)	0.00
Female	2,079 (28.5)	20,200 (28.5)	0.00
**Age**			
Mean (SD)	27.39 (10.9)	27.44 (10.9)	0.00
14-18 years	1,574 (21.6)	15,218 (21.5)	0.00
19-24 years	2,165 (29.7)	21,087 (29.7)	0.00
25-44 years	2,846 (39.1)	27,720 (39.1)	0.00
45-65 years	700 (9.6)	6,901 (9.7)	0.00
**Rurality**			
Urban	6,432 (88.3)	63,410 (89.4)	0.04
Rural	778 (10.7)	7,216 (10.2)	0.02
Missing	75 (1.0)	290 (0.4)	0.07
**Homelessness**			
Yes	844 (11.6)	180 (0.3)	0.49
No	6,441 (88.4)	70,736 (99.7)	0.49
**Neighbourhood Income Quintile**			
1 (poorest)	2,100 (28.8)	13,816 (19.5)	0.22
2	1,514 (20.8)	13,748 (19.4)	0.04
3	1,271 (17.4)	14,091 (19.9)	0.06
4	1,189 (16.3)	14,476 (20.4)	0.11
5 (richest)	1,115 (15.3)	14,391 (20.3)	0.13
Missing	96 (1.3)	394 (0.6)	0.08
**Long Term Resident of Canada**			
Yes	6,699 (92.0)	59,149 (83.4)	0.26
No	586 (8.0)	11,767 (16.6)	0.26
**Acute Care (ED or Hospital) Substance Use Visits in Past 5 Years**			
Any	4,512 (61.9)	2,466 (3.5)	1.59
Alcohol	2,253 (30.9)	1,673 (2.4)	0.83
Cannabis	1,690 (23.2)	490 (0.7)	0.74
Cocaine	1,263 (17.3)	217 (0.3)	0.63
Amphetamines	1,191 (16.3)	137 (0.2)	0.61
Opioids	1,357 (18.6)	271 (0.4)	0.65
Other drug use	502 (6.9)	63 (0.1)	0.38
**Acute Care (ED or Hospital) Mental Health Visits in Past 5 Years**			
Any	3,572 (49.0)	3,421 (4.8)	1.15
Mood Disorder	1,299 (17.8)	1,145 (1.6)	0.58
Anxiety Disorder	1,872 (25.7)	1,992 (2.8)	0.69
Schizophrenia	918 (12.6)	341 (0.5)	0.51
Deliberate Self harm	1,579 (21.7)	576 (0.8)	0.70
Other	717 (9.8)	487 (0.7)	0.42
**Outpatient Mental Health and Substance Visits in Past 5 Years**			
Any	5,622 (77.2)	23,544 (33.2)	0.99
Family Physician	5,382 (73.9)	22,783 (32.1)	0.92
Psychiatrist	3,169 (43.5)	5,578 (7.9)	0.89
**Any Acute or Outpatient Mental Health or Substance Visit in Past 5 Years**			
Yes	6,448 (88.5)	24,660 (34.8)	1.33
No	837 (11.5)	46,256 (65.2)	1.33

***Note.*** ED = Emergency department. SD = Standard deviation.

^A^Sum to more than 100% as individuals could have more than one hallucinogen code on presentation.

^B^Codes in designated mental health beds are either for hallucinogen dependence or abuse.

**Fig 1:**
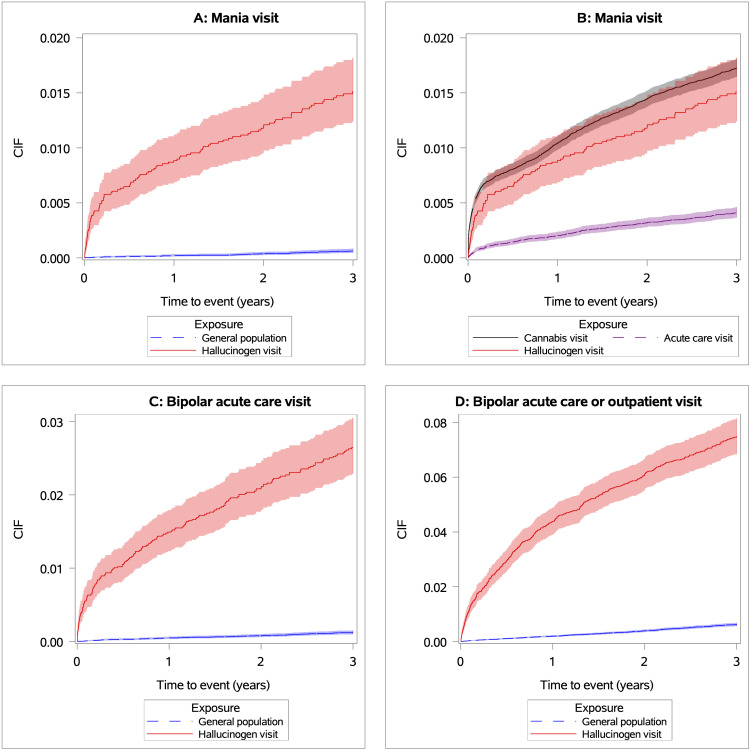
Cumulative incidence function (CIF) curves comparing the risk of mania and bipolar disorder acute care over three years. *Panel A* shows the incidence of mania acute care for individuals with acute care involving hallucinogens compared to the matched general population. *Panel B* shows the secondary analyses of the incidence for individuals with acute care involving hallucinogens compared to the matched population of those with all-cause acute care (excluding hallucinogen-relate visits) and those with cannabis-related acute care. *Panel C* shows the incidence of bipolar disorder acute care, and *Panel D* shows the risk of bipolar disorder in an acute care or outpatient setting for individuals with acute care involving hallucinogens compared to the matched general population. Shaded regions represent 95% Confidence Intervals.

Cumulative incidence functions for the risk of mania or BD over time are presented in **Fig 1**.

Within three years, 104 (1.43%) individuals with acute care involving hallucinogens compared to 41 (0.06%) members of the matched general population received acute care for mania – a 25-fold increase in risk (**Table 2**). After overlap weighting, having an acute care visit involving hallucinogens was associated with a 6-fold greater risk (hazard ratio [HR] 5.97, 95% CI 3.29, 10.82) of acute care involving mania within 3-years, relative to individuals without acute care for hallucinogens who otherwise had the same sociodemographic, and history of prior substance use or mental healthcare. A sensitivity analysis excluding individuals with depressive symptoms yielded similar results (HR 5.81, 95%CI 2.84, 11.88), see **Table 2**.

**Table 2:** Risk of mania in individuals with acute care involving hallucinogens compared to the general population or individuals with acute care for other substances.

	No. at risk	Mania diagnosis^a^	Mania diagnosis 1 year	Mania diagnosis 3 years	Mania diagnosis 5 years	Crude rate per 100,000^B^	Age and sex overlap weighted HR (95%CI)^B^	Overlap weighted HR (95%CI)^B,C^
			No. (%)					
**Comparator 1: General Population**								
Acute Care Visit Involving Hallucinogens	7,285	148	64 (0.88)	104 (1.43)	126 (1.73)	525.61	25.16 (17.53, 36.10)	5.97 (3.29, 10.82)
General Population	70,916	114	15 (0.02)	41 (0.06)	69 (0.10)	20.72	Ref.	Ref.
**Comparator 2: All-cause Acute Care** ^**D**^								
Acute Care Visit Involving Hallucinogens	7,285	148	64 (0.88)	104 (1.43)	126 (1.73)	525.61	3.71 (2.96, 4.65)	2.43 (1.88, 3.15)
Acute Care Visits for Reason other than Hallucinogens	72,847	453	147 (0.20)	284 (0.39)	358 (0.49)	140.84	Ref.	Ref.
**Comparator 3: Acute Care for Cannabis**								
Acute Care Visit Involving Hallucinogens	7,285	148	64 (0.88)	104 (1.43)	126 (1.73)	525.61	0.85 (0.70, 1.03)	0.93 (0.76, 1.13)
Acute Care visit Involving Cannabis	102,931	2,409	1,071 (1.04)	1,705 (1.66)	2,045 (1.99)	606.09	Ref.	Ref.
**Comparator 1: General Pop. (Sensitivity Analysis, No Prior Major Depression)**								
Acute Care Visit Involving Hallucinogens	5,986	96	37 (0.62)	64 (1.07)	79 (1.32)	391.28	22.32 (14.72, 33.83)	5.81 (2.84, 11.88)
General Population	69,771	99	Suppressed^E^	34 (0.05)	58 (0.08)	17.46	Ref.	Ref.

***Note.*** HR = Hazard ratio. CI = Confidence interval.

^A^Diagnosis over maximum follow up period available

^B^Rate or hazard ratios at 3-years of follow up.

^C^Weighted for age, sex, neighbourhood income quintile, rurality, immigration status, homelessness, past five-year outpatient, ED, and hospital-based care for mental health (anxiety, mood disorder, self-harm, schizophrenia, and other) and substance use (alcohol, stimulants, cannabis, opioids, amphetamines, poly-substance, other).

^D^Excludes hallucinogen-related visits.

^E^Supressed to comply with privacy requirements at ICES.

Acute care involving hallucinogens was also associated with an elevated risk for incident acute care for BD (HR 3.75, 95% CI 2.49, 5.65) or incident care in an outpatient or acute care setting for BD (HR 3.50 95% CI 2.85, 4.31) relative to individuals without acute care for hallucinogens with similarly weighted sociodemographic and mental healthcare history, see **Table 3**.

**Table 3:** Risk of bipolar disorder (BD) among individuals with acute care involving hallucinogens or incident care in an outpatient or acute care setting relative to the general population.

	No. at risk	BD diagnosis^a^	BD diagnosis 1 year	BD diagnosis 3 years	BD diagnosis 5 years	Crude rate per 100,000^B^	Age and sex overlap weighted HR (95%CI)^B^	Overlap weighted HR (95%CI)^B,C^
			No. (%)					
**Acute Care Setting Only**								
Visit Involving Hallucinogens	7,285	269	108 (1.48)	182 (2.50)	228 (3.13)	926.5	22.70 (17.45, 29.52)	3.75 (2.49, 5.65)
General Population	70,916	194	34 (0.05)	80 (0.11)	124 (0.17)	40.43	Ref.	Ref.
**Acute Care or Outpatient Setting**								
Visit Involving Hallucinogens	6,772	720	294 (4.34)	481 (7.10)	579 (8.55)	2,720.61	12.98 (11.37, 14.81)	3.50 (2.85, 4.31)
General Population	70,244	919	135 (0.19)	403 (0.58)	602 (0.86)	206.09	Ref.	Ref.

***Note.*** HR = Hazard ratio. CI = Confidence interval.

^A^Diagnosis over maximum follow up period available.

^B^Rate or hazard ratios at 3-years of follow up.

^C^Weighted for age, sex, neighbourhood income quintile, rurality, immigration status, homelessness, past five-year outpatient, ED, and hospital-based care for mental health (anxiety, mood disorder, self-harm, schizophrenia, and other) and substance use (alcohol, stimulants, cannabis, opioids, amphetamines, poly-substance use, other).

The secondary analysis comparing individuals with acute care involving hallucinogens to those with all-cause acute care (excluding hallucinogen visits) included 80,132 individuals. See **Table B in** S1 Text for cohort characteristics and **Table C in** S1 Text for cohort characteristics before and after propensity matching. Within three years, 104 (1.4%) individuals with acute care involving hallucinogens compared to 284 (0.4%) individuals with all-cause acute care, had acute care for mania – a 3.7-fold increase in risk (see **Table 2**). After overlap weighting for sociodemographics and prior substance use or mental healthcare, acute care involving hallucinogens was associated with a 2.4-fold greater risk (HR 2.43, 95% CI 1.88, 3.15) of acute care involving mania within 3-years was, relative to acute care visits for other reasons. Sensitivity analyses requiring only one ED visit for mania rather than two increased the number of individuals with incident mania and showed consistent associations (HR 5.88, 95% CI 3.60, 9.60) between acute care due to hallucinogens and greater risk of incident mania (**see Table D in** S1 Text). Sensitivity analyses excluding data from COVID-19 showed similar associations (HR 6.94, 95%CI 3.33, 14.46) compared to the primary analysis (see **Table D in** S1 Text).

The secondary analysis comparing individuals with acute care involving hallucinogens to acute care involving cannabis included 110,216 individuals. See **Table B in** S1 Text for cohort characteristics and **Table E in** S1 Text for cohort characteristics before and after propensity matching. Within three years, 104 (1.4%) individuals with acute care involving hallucinogens compared to 1,705 (1.7%) individuals with acute care involving cannabis had acute care for mania. After overlap weighting for sociodemographics, and prior substance use or mental healthcare, the risk of subsequent acute care involving mania did not differ significantly between individuals with acute care involving hallucinogens relative to cannabis (HR 0.93, 95% CI 0.76, 1.13).

Age stratified analysis showed similar elevations in risk of incident acute care for mania associated with acute care for hallucinogens relative to the general population for individuals aged 14–24 compared to individuals aged 25–65. Sex stratified analysis found larger elevations in risk of incident acute care for mania associated with acute care for hallucinogens relative to the general population for males (HR 7.54, 95%CI 3.55, 16.01) relative to females (HR 3.50, 95%CI 1.28, 9.55), though the difference was not statistically significant. See **Table F in** S1 Text for details.

## Discussion

In this population-based study of 9.3 million people, we found that hallucinogen-related acute care was associated with a 25-fold higher risk of an incident episode of mania requiring acute care compared to individuals of the same age and sex in the general population (3-year incident diagnosis of 1.43% versus 0.06%). Risk of mania rose most rapidly in the period immediately following the acute care for substance use. After controlling for confounding factors through weighting, acute care visits for hallucinogens were associated with a 6-fold higher risk of acute care for mania compared to individuals without acute care for hallucinogens who had otherwise similar sociodemographic and past mental healthcare service use. Those with hallucinogen-related acute care visits were also more likely to have incident acute- or outpatient care for BD. Acute care involving hallucinogens was not associated with a significantly different risk of subsequent mania-related acute care compared to cannabis related acute care, although the associated risk remained higher relative to all-cause acute care unrelated to hallucinogen use.

Hallucinogen use for both recreational and medical purposes is growing in popularity, however, evidence of its safety beyond controlled clinical settings and its association with mania and BD remains limited [13]. Contemporary trials have begun to include individuals with a diagnosis of bipolar II disorder (BD II) and have reported improvements in depressive symptoms [36,37]. However, these studies acknowledge limitations in the generalizability of their findings due to small sample size, self-reported responses, and caution against extrapolating findings to individuals with a diagnosis of BD I [36,37]. A recently published study including 505 people who used psilocybin over the study period found that people who self-reported psilocybin use in a non-medical setting reported worsening severity of mania symptoms [17]. Our study is consistent with these prior findings and novel population-level insight into hallucinogen use that required acute care for mania and BD.

Given the wide range of potential hallucinogens captured in the administrative health diagnoses, we are unable to identify the underlying factors driving the observed association with increased risk of bipolar disorder. Hallucinogens, including ketamine, MDMA and psilocybin, are increasingly being examined as a treatment for mental health disorders and are broadly though to work by altering neurotransmitters and increasing neuroplasticity [38]. It is possible that changes in neurotransmitters and neuroplasticity arising from use of both dissociative and serotonergic hallucinogens could contribute to or be associated with the occurance of hypomanic and manic episodes [39–43]. In our study, two-thirds of individuals with acute care related to hallucinogen use had prior acute care visits for substance use and nearly half had a prior mental health related acute care visit. These findings align with existing literature which suggests that factors including family history, prior diagnosis of mental health conditions, polysubstance use, and frequency of use, may increase the risk of adverse psychiatric symptoms following hallucinogen use [13,44,45].

Throughout our study period a very limited number of individuals would have had access to hallucinogens in a clinical trial setting or via healthcare provider prescription. Prior research has highlighted the widespread availability of gray market physical and online retailers selling hallucinogens in Canada [46]. Consequently, the vast majority of our sample was likely exposed to hallucinogens through recreational use. As clinical trials may not detect relatively rare events such as mania, or reflect risks in naturalistic settings, ongoing monitoring of risks associated with hallucinogens is indicated both in trials and observational research. Collectively, in order to balance the potential benefits of therapeutic use of hallucinogens against possible risks of large increases in both therapeutic and recreational use of hallucinogens, better information is needed about the overall risks of adverse events, including mania, along with whether certain individuals are at greater risk.

Our study has limitations. First, our research examined individuals who required urgent medical attention following hallucinogen use and the observed association with an increased risk of mania and bipolar disorder should not be inferred to apply to the vast majority of individuals who use hallucinogens and do not require urgent healthcare. Second, detailed data on patterns of use (e.g., frequency, dosing) or the type of hallucinogen (e.g., serotonergic hallucinogens, ketamine, PCP) used were not available. Consequently, the results of this study may not apply to all types of hallucinogens. Third, although the exposure captures a clinically relevant pattern of hallucinogen use requiring ED or hospital-based care, the hallucinogen-related diagnostic codes used to capture our exposure have not been chart validated, and therefore some visits may be misclassified or missed. Fourth, while we accounted for a robust set of potential confounders, including sociodemographic factors, history of mental disorder, substance use disorder, and homelessness individuals with acute care involving hallucinogens likely differed further from the general population on unmeasured confounders [47]. Potential unmeasured confounders include individual-level income, educational attainment, family history of mental health disorders, adverse childhood experiences, and genetics, and could be driving part of the observed association and the magnitude of the observed associations should be interpreted with caution. Fifth, our outcome was based on having received healthcare and therefore, manic syndromes not identified through receipt of care, which likely capture more mild presentations, would not have been included. Lastly, while our analyses exclude individuals with established BD diagnosis part of the observed association could be driven by reverse causation (e.g., use of hallucinogens to self-medicate symptoms of BD that have not yet been diagnosed). Part of the findings may reflect improved detection of existing mania or BD based on referrals following the ED visit involving hallucinogen use. Further prospective studies with information on the temporality of symptoms rather than receipt of care are indicated.

We found that acute care for hallucinogen use is associated with a substantially elevated risk of incident mania and bipolar disorder. Despite a resurgence of interest and use of hallucinogens both in therapeutic and recreational contexts, there are major gaps in our understanding of associated risks beyond the duration of a randomized controlled trial, and in representative populations. Further research on the underlying risk associated with different types and patterns of hallucinogen use along with the distinct mechanisms possibly leading to adverse mental health outcomes is needed.

## Supporting information

S1 File RECORD checklist. (DOCX)

S1 Text Supplement methods, tables and figures. (DOCX)
