# Supplementary material for: Hospital-based care for hallucinogens and risk of mania and bipolar disorder: A population-based cohort study
Source: PLoS Med. 2025 Dec 2;22(12):e1004805. doi: 10.1371/journal.pmed.1004805 (PMC12671790; doi:10.1371/journal.pmed.1004805)
Supplement: S1 Text — (DOCX) [file pmed.1004805.s002.docx]

**S1 Text: Supporting Information**

**Title:** Hospital-based care for hallucinogens and risk of mania and bipolar disorder: A population-based cohort study

**Authors:** Daniel T. Myran, Rachael MacDonald-Spracklin, Michael Pugliese, Maya Gibb, Jess G. Fiedorowicz, Tyler S. Kaster, MD, and Marco Solmi.

Table of Contents

[Methods A. Index dates assignment and list of diagnostic codes to identify the development of mania or bipolar disorder 2](#_Toc212801127)

[Methods B. List of diagnostic codes used to identify non-hallucinogen ED visits 4](#_Toc212801128)

[Methods C. Covariate definition codes 5](#_Toc212801129)

[Figure A. Cohort flow of participant exclusions for the primary outcome mania between hallucinogen acute care visits, vs the general population 6](#_Toc212801130)

[Table A. Covariate balance before and after overlap weighting, primary outcome mania between hallucinogen acute care visits, vs the general population 7](#_Toc212801131)

[Table B. Characteristics of individuals within the all-cause acute care and cannabis acute care cohorts 9](#_Toc212801132)

[Table C. Covariate balance before and after overlap weighting, all-cause acute care secondary analysis 12](#_Toc212801133)

[Table D. Risk of mania in individuals with acute care involving hallucinogens compared to the general population, sensitivity analysis excluding COVID-19 years and requiring only 1 ED visit for a mania diagnosis 14](#_Toc212801134)

[Table E. Covariate balance before and after overlap weighting, cannabis visit secondary analysis 15](#_Toc212801135)

[Table F. Risk of mania in individuals with acute care involving hallucinogens compared to the general population, sensitivity analysis stratified by age and sex 17](#_Toc212801136)

# Methods A. Index dates assignment and list of diagnostic codes to identify the development of mania or bipolar disorder

For the general population we assigned index dates that matched the distribution of index dates from individuals with acute care involving hallucinogens. This was completed in the following steps.

1. We captured the index date of acute care involving hallucinogens for each individual in the hallucinogen exposure group
2. Each individual with acute care involving hallucinogen and their index data were given a unique identification number
3. Each individual in the general population was randomly assigned one of the unique identification numbers from the acute care involving hallucinogen group
4. Individuals in the general population received the index data from the unique identification number to which they were randomly assigned.

The index date for matched individuals with incident all-cause acute care, or individuals with acute care due to cannabis was the date of the all-cause acute care or cannabis care.

| **Condition** | **Corresponding Diagnostic Codes** |
| --- | --- |
| **Manic Episode** | **ICD-10 Codes** |
| Hypomania | F30.0 |
| Mania with or without Psychotic Symptoms | F30.1, F30.2 |
| Other Manic Episodes or Unspecified Manic Episode | F30.8, F30.9 |
| **Bipolar Affective Disorder** | **ICD-10 Codes** |
| Bipolar Affective Disorder, current episode hypomanic | F31.0 |
| Bipolar Affective Disorder, current episode manic (with, without psychotic symptoms or mixed) | F31.1, F31.2, 31.6 |
| **Bipolar 1 Disorder** | **ICD-9/DSM Codes** |
| Bipolar 1 Disorder, single manic episode | F296.0x |
| Bipolar 1 Disorder, most recent episode manic | F296.4x |
| Bipolar 1 Disorder, most recent episode mixed | F296.6x |
| Bipolar 1 Disorder, most recent episode unspecified | F296.7x |
| Bipolar 1 Disorder NOS or Bipolar 2 Disorder | F296.8x |

***Note.*** ICD = International Classification of Diseases. DSM = Diagnostic and Statistical Manual of Mental Disorders. NOS = Not otherwise specified.

#

# Methods B. List of diagnostic codes used to identify non-hallucinogen ED visits

Cannabis Codes:

We identified an acute care visit due to cannabis using *International Classification of Diseases*, *10th Revision* (*ICD-10*) codes, when listed as either the main or contributing reason for the visit. The full list of codes can be found in the table below.

| **Condition** | **ICD-9-CM/ICD-10-CM (OMHRS)^1^** | **ICD-10-CA (DAD)^1^** |
| --- | --- | --- |
| Acute cannabis intoxication | F12.0 | F12.0 |
| Harmful use | N/A | F12.1 |
| **Cannabis Use Disorder** |  |  |
| Dependence | 304.3 / F12.2 | F12.2 |
| Withdrawal | F12.3, F12.4 | F12.3, F12.4 |
| Cannabis-induced psychosis | F12.5 or F12.7 | F12.5 or F12.7 |
| Other cannabis events | F12.6, F12.8, F12.9 | F12.6, F12.8, F12.9 |
| Cannabis poisoning | T40.7 | T40.7 |

***Note.*** OMHRS = Ontario mental health reporting system. DAD = Discharge abstract database.

Other Visit Codes:

Other visit codes were included for all other events including ED visits, hospitalizations and mental health hospitalizations, excluding any visit with hallucinogens involved. We then matched all-cause acute care visits at a 10:1 ratio to hallucinogen related visits.

# Methods C. Covariate definition codes

- Recent immigration status was determined using the Immigration Refugees and Citizenship Canada’s Permanent Resident Database, which identifies all individuals who arrived in Ontario between 1985 and 2020.
- Documented homelessness was determined using a validated algorithm where homelessness is identified and documented during hospital encounters.^1^
- Urban or rural residence and neighbourhood-level income quintiles were defined using Statistics Canada census data.^2^
- Mental health emergency department (ED) visit or hospitalization in past five years and subtypes were defined using codes from Mental Health Codes from: Mental Health and Addictions Scorecard and Evaluation Framework indicator.^3^
- Substance use disorder ED visit or hospitalization in past five years and subtypes were defined using codes from Mental Health Codes from: Mental Health and Addictions Scorecard and Evaluation Framework indicator.^3^
- Outpatient substance use or mental health visits were defined using diagnostic and billing codes from Mental Health and Addictions Scorecard and Evaluation Framework indicator.^3^

**References**

1. Richard L, Carter B, Nisenbaum R, Liu M, Hwang SW. Identification of homelessness using health administrative data in Ontario, Canada following a national coding mandate: a validation study. *J Clin Epidemiol*. 2024;172:111430. doi:10.1016/j.jclinepi.2024.111430

2. Statistics Canada. *Population Centre and Rural Area Classification*.; 2016.

3. ICES. Mental Health and Addictions System Performance in Ontario: A Baseline Scorecard. Accessed June 2, 2024. https://www.ices.on.ca/publications/research-reports/mental-health-and-addictions-system-performance-in-ontario-a-baseline-scorecard/

4. Van Walraven C, Austin PC, Jennings A, Quan H, Forster AJ. A modification of the Elixhauser comorbidity measures into a point system for hospital death using administrative data. *Med Care*. 2009;47(6):626-633. doi:10.1097/MLR.0B013E31819432E5

5. Quan H, Sundararajan V, Halfon P, et al. Coding algorithms for defining comorbidities in ICD-9-CM and ICD-10 administrative data. *Med Care*. 2005;43(11):1130-1139. doi:10.1097/01.MLR.0000182534.19832.83

6. Koné Pefoyo AJ, Bronskill SE, Gruneir A, et al. The increasing burden and complexity of multimorbidity disease epidemiology - Chronic. *BMC Public Health*. 2015;15(1):1-11. doi:10.1186/S12889-015-1733-2/TABLES/3

# Figure A. Cohort flow of participant exclusions for the primary outcome mania between hallucinogen acute care visits, vs the general population


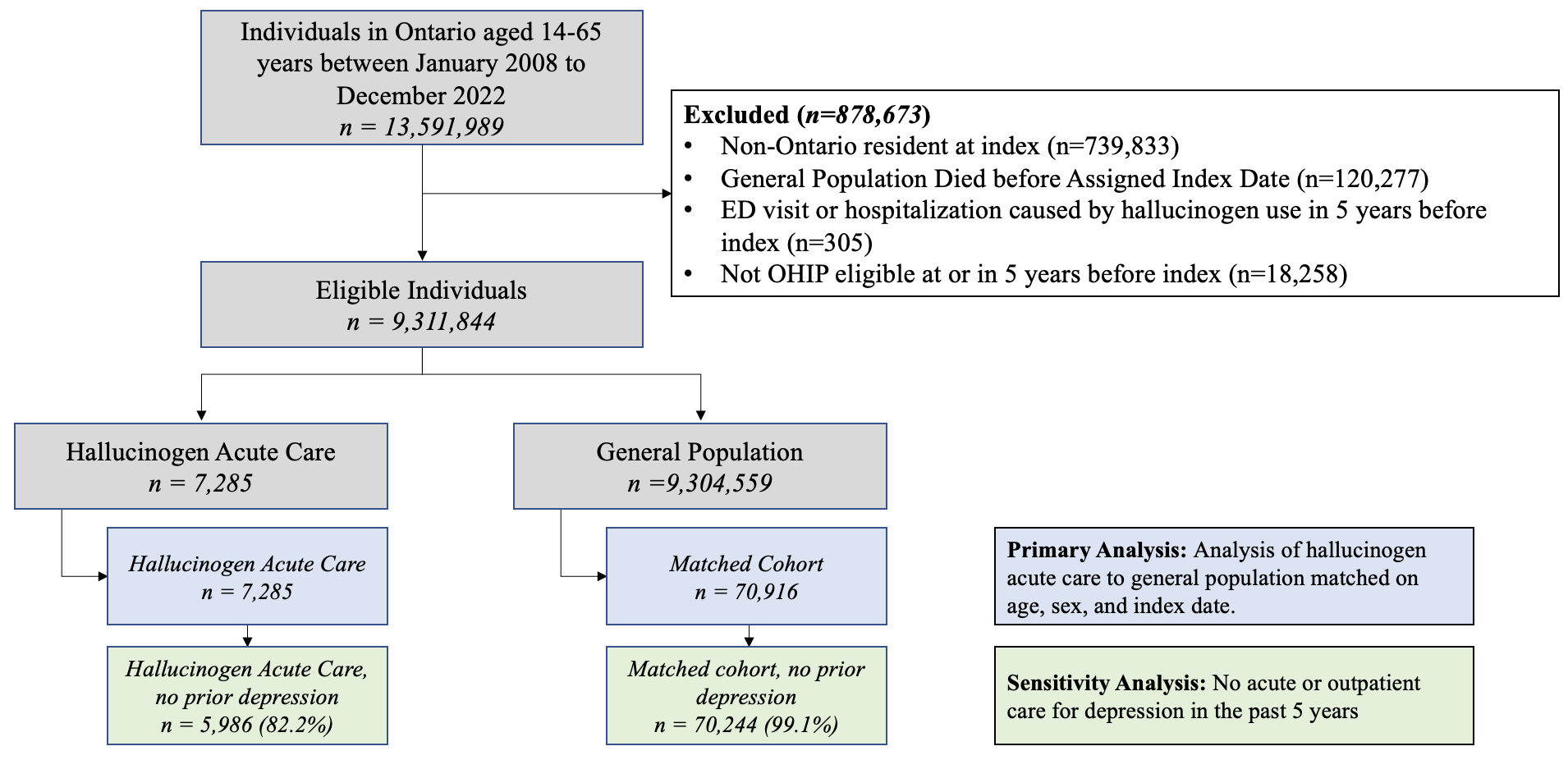


***Note.*** ED = Emergency department.

# Table A. Covariate balance before and after overlap weighting, primary outcome mania between hallucinogen acute care visits, vs the general population

| **Variable** | **Unweighted Hallucinogen Acute Care** | **Unweighted Other Acute Care** | **Unweighted SMD** | **Weighted Hallucinogen Acute Care** | **Weighted Other Acute Care** | **Weighted SMD** |
| --- | --- | --- | --- | --- | --- | --- |
| **Overall** | 7,285 | 70,916 | . | 3,896.2 | 3,896.2 | . |
| **Sex** |  |  |  |  |  |  |
| Female | 28.54% | 28.48% | 0.001 | 27.53% | 27.53% | 0.000 |
| **Age** |  |  |  |  |  |  |
| Mean | 27.39 | 27.44 | 0.004 | 26.52 | 26.52 | 0.000 |
| **Income Quintile** |  |  |  |  |  |  |
| Q1 | 28.83% | 19.48% | 0.220 | 25.36% | 25.36% | 0.000 |
| Q2 | 20.78% | 19.39% | 0.035 | 20.84% | 20.84% | 0.000 |
| Q3 | 17.45% | 19.87% | 0.062 | 18.26% | 18.26% | 0.000 |
| Q4 | 16.32% | 20.41% | 0.106 | 17.77% | 17.77% | 0.000 |
| Q5 | 15.31% | 20.29% | 0.131 | 16.90% | 16.90% | 0.000 |
| Missing | 1.32% | 0.56% | 0.079 | 0.87% | 0.87% | 0.000 |
| **Rurality** |  |  |  |  |  |  |
| Rural | 10.68% | 10.18% | 0.016 | 10.97% | 10.97% | 0.000 |
| Urban | 88.29% | 89.42% | 0.036 | 88.39% | 88.39% | 0.000 |
| Missing | 1.03% | 0.41% | 0.073 | 0.65% | 0.65% | 0.000 |
| **Long Term Resident of Canada** | |  |  |  |  |  |
| Yes | 8.04% | 16.59% | 0.262 | 9.90% | 9.90% | 0.000 |
| **Document History of Homelessness** | |  |  |  |  |  |
| Yes | 11.59% | 0.25% | 0.495 | 2.82% | 2.82% | 0.000 |
| **Substance Use Acute Care Visits in Past 5 Years** | | |  |  |  |  |
| Alcohol | 30.93% | 2.36% | 0.830 | 15.33% | 15.33% | 0.000 |
| Cannabis | 23.20% | 0.69% | 0.740 | 8.38% | 8.38% | 0.000 |
| Cocaine | 17.34% | 0.31% | 0.630 | 4.05% | 4.05% | 0.000 |
| Amphetamines | 16.35% | 0.19% | 0.614 | 3.03% | 3.03% | 0.000 |
| Opioids | 18.63% | 0.38% | 0.655 | 4.69% | 4.69% | 0.000 |
| Polysubstance | 26.73% | 0.60% | 0.822 | 7.13% | 7.13% | 0.000 |
| Other | 6.89% | 0.09% | 0.377 | 1.29% | 1.29% | 0.000 |
| **Mental Health Acute Care Visits in Past 5 Years** | | |  |  |  |  |
| Mood | 17.83% | 1.61% | 0.569 | 8.62% | 8.62% | 0.000 |
| Anxiety | 25.70% | 2.81% | 0.693 | 13.00% | 13.00% | 0.000 |
| Schizophrenia | 12.60% | 0.48% | 0.506 | 4.40% | 4.40% | 0.000 |
| Self-Harm | 21.67% | 0.81% | 0.700 | 8.48% | 8.48% | 0.000 |
| Other | 9.84% | 0.69% | 0.419 | 3.82% | 3.82% | 0.000 |
| **Outpatient Mental Health and Addiction Visits in Past 5 Years** | | | |  |  |  |
| Family medicine | 73.88% | 32.13% | 0.921 | 61.43% | 61.43% | 0.000 |
| Psychiatry | 43.50% | 7.87% | 0.893 | 26.84% | 26.84% | 0.000 |

***Note.*** SMD = Standardized mean difference.

# Table B. Characteristics of individuals within the all-cause acute care and cannabis acute care cohorts

|  | **All-Cause Acute Care (n=72,847)** | **Cannabis Acute Care (n=102,931)** |
| --- | --- | --- |
|  | **N (%)** | |
| **Location of Acute Care** |  |  |
| Emergency Department | 62,280 (85.5) | 76,969 (74.8) |
| Acute Care Hospital Bed | 4,740 (6.5) | 9,010 (8.8) |
| Specialized Mental Health Hospital Bed | 5,827 (8.0) | 16,952 (16.5) |
| **Sex** |  |  |
| Male | 52,057 (71.5) | 63,869 (62.1) |
| Female | 20,790 (28.5) | 39,062 (37.9) |
| **Age** |  |  |
| Mean ± SD | 27.39 (10.89) | 28.81 (12.55) |
| 14-18 years | 15,737 (21.6) | 22,513 (21.9) |
| 19-24 years | 21,650 (29.7) | 28,377 (27.6) |
| 25-44 years | 28,460 (39.1) | 37,110 (36.1) |
| 45-64 years | 7,000 (9.6) | 14,931 (14.5) |
| **Rurality** |  |  |
| Urban | 59,968 (82.3) | 90,320 (87.7) |
| Rural | 12,576 (17.3) | 12,036 (11.7) |
| **Homelessness** |  |  |
| Yes | 2,928 (4.0) | 6,594 (6.4) |
| No | 69,919 (96.0) | 96,337 (93.6) |
| **Neighbourhood Income Quintile** |  |  |
| 1 (poorest) | 18,201 (25.0) | 29,781 (28.9) |
| 2 | 15,006 (20.6) | 21,708 (21.1) |
| 3 | 14,008 (19.2) | 18,492 (18.0) |
| 4 | 13,184 (18.1) | 16,802 (16.3) |
| 5 (richest) | 11,970 (16.4) | 15,346 (14.9) |
| **Long Term Resident of Canada** |  |  |
| Yes | 65,487 (89.9) | 94,122 (91.4%) |
| No | 7,360 (10.1) | 8,809 (8.6%) |
| **Acute Care (ED or Hospital) Substance Use Visits in Past 5 Years** | | |
| Any | 12,669 (17.4) | 41,172 (40.0) |
| Alcohol | 7,499 (10.3) | 26,574 (25.8) |
| Cannabis | 3,996 (5.5) | 102,931 (100.0) |
| Cocaine | 2,055 (2.8) | 10,516 (10.2) |
| Amphetamines | 1,903 (2.6) | 6,301 (6.1) |
| Opioids | 2,642 (3.6) | 8,763 (8.5) |
| Other drug use | 815 (1.1) | 2,977 (2.9) |
| Polysubstance Use | 4,298 (5.9) | 11,973 (11.6) |
| **Acute Care (ED or Hospital) Mental Health Visits in Past 5 Years** | | |
| Any | 18,948 (26.0) | 52,664 (51.2) |
| Mood Disorder | 7,998 (11.0) | 21,437 (20.8) |
| Anxiety Disorder | 11,540 (15.8) | 26,230 (25.5) |
| Schizophrenia | 4,870 (6.7) | 13,974 (13.6) |
| Deliberate Self harm | 4,432 (6.1) | 12,749 (12.4) |
| Other | 4,341 (6.0) | 8,863 (8.6) |
| **Outpatient Mental Health or Substance Use Visit in Past 5 Years** | | |
| Any | 39,444 (54.1) | 77,147 (75.0) |
| Family Physician | 37,750 (51.8) | 72,768 (70.7) |
| Psychiatrist | 16,679 (22.9) | 42,400 (41.2) |
| **Any Acute or Outpatient Visit for Mental Health or Substance Use in Past 5 Years** | | |
| Yes | 42,987 (59.0) | 87,826 (85.3) |
| No | 29,860 (41.0) | 15,105 (14.7) |

***Note.*** SD = Standard deviation. ED = Emergency department.

^A^Sums to more than 100% as individuals could have more than one cannabis code on presentation

# Table C. Covariate balance before and after overlap weighting, all-cause acute care secondary analysis

| **Variable** | **Unweighted Hallucinogen Acute Care** | **Unweighted Other Acute Care** | **Unweighted SMD** | **Weighted Hallucinogen Acute Care** | **Weighted Other Acute Care** | **Weighted SMD** |
| --- | --- | --- | --- | --- | --- | --- |
| **Overall** | 7,285 | 72,847 | . | 5874.2 | 5874.2 | . |
| **Sex** |  |  |  |  |  |  |
| Female | 28.54% | 28.54% | 0.000 | 28.40% | 28.40% | 0.000 |
| **Age** |  |  |  |  |  |  |
| Mean | 27.39 | 27.39 | 0.000 | 27.23 | 27.23 | 0.000 |
| **Income Quintile** |  |  |  |  |  |  |
| Q1 | 28.83% | 24.99% | 0.087 | 28.19% | 28.19% | 0.000 |
| Q2 | 20.78% | 20.60% | 0.005 | 20.93% | 20.93% | 0.000 |
| Q3 | 17.45% | 19.23% | 0.046 | 17.64% | 17.64% | 0.000 |
| Q4 | 16.32% | 18.10% | 0.047 | 16.64% | 16.64% | 0.000 |
| Q5 | 15.31% | 16.43% | 0.031 | 15.42% | 15.42% | 0.000 |
| Missing | 1.32% | 0.66% | 0.067 | 1.17% | 1.17% | 0.000 |
| **Rurality** |  |  |  |  |  |  |
| Rural | 10.68% | 17.26% | 0.191 | 11.45% | 11.45% | 0.000 |
| Urban | 88.29% | 82.32% | 0.169 | 87.68% | 87.68% | 0.000 |
| Missing | 1.03% | 0.42% | 0.072 | 0.88% | 0.88% | 0.000 |
| **Long Term Resident of Canada** | |  |  |  |  |  |
| Yes | 8.04% | 10.10% | 0.072 | 8.45% | 8.45% | 0.000 |
| **Document History of Homelessness** | |  |  |  |  |  |
| Yes | 11.59% | 4.02% | 0.285 | 10.03% | 10.03% | 0.000 |
| **Substance Use Acute Care Visits in Past 5 Years** | | |  |  |  |  |
| Alcohol | 30.93% | 10.29% | 0.528 | 26.13% | 26.13% | 0.000 |
| Cannabis | 23.20% | 5.49% | 0.522 | 18.47% | 18.47% | 0.000 |
| Cocaine | 17.34% | 2.82% | 0.497 | 12.51% | 12.51% | 0.000 |
| Amphetamines | 16.35% | 2.61% | 0.482 | 11.89% | 11.89% | 0.000 |
| Opioids | 18.63% | 3.63% | 0.491 | 14.10% | 14.10% | 0.000 |
| Polysubstance | 26.73% | 5.90% | 0.587 | 20.77% | 20.77% | 0.000 |
| Other | 6.89% | 1.12% | 0.298 | 4.75% | 4.75% | 0.000 |
| **Mental Health Acute Care Visits in Past 5 Years** | | |  |  |  |  |
| Mood | 17.83% | 10.98% | 0.196 | 16.57% | 16.57% | 0.000 |
| Anxiety | 25.70% | 15.84% | 0.245 | 23.73% | 23.73% | 0.000 |
| Schizophrenia | 12.60% | 6.69% | 0.201 | 11.72% | 11.72% | 0.000 |
| Self-Harm | 21.67% | 6.08% | 0.463 | 17.63% | 17.63% | 0.000 |
| Other | 9.84% | 5.96% | 0.144 | 9.04% | 9.04% | 0.000 |
| **Outpatient Mental Health and Addiction Visits in Past 5 Years** | | | |  |  |  |
| Family Medicine | 73.88% | 51.82% | 0.469 | 70.60% | 70.60% | 0.000 |
| Psychiatry | 43.50% | 22.90% | 0.448 | 39.40% | 39.40% | 0.000 |

***Note.*** SMD = Standardized mean difference.

# Table D. Risk of mania in individuals with acute care involving hallucinogens compared to the general population, sensitivity analysis excluding COVID-19 years and requiring only 1 ED visit for a mania diagnosis

|  | **No. at Risk** | **Mania Diagnosis^A^** | **Mania Diagnosis**  **1 Year** | **Mania Diagnosis**  **3 Years** | **Mania Diagnosis**  **5 Years** | **Crude Rate^B^** | **Age and Sex Overlap Weighted HR (95%CI)^B^** | **Overlap Weighted HR (95%CI)^B,C^** |
| --- | --- | --- | --- | --- | --- | --- | --- | --- |
| ***Exclusion of COVID-19 Period*** | |  |  | **No. (%)** |  |  |  |  |
| **Comparator 1: General Population** | |  |  | | |  |  |  |
| Acute Care Visit Involving Hallucinogens | 4,720 | 92 | 47 (1.00) | 71 (1.50) | 85 (1.80) | 560.69 | 22.50 (14.76, 14.46) | 6.94 (3.33, 14.46) |
| General Population | 45,595 | 66 | Suppressed^D^ | 31 (0.07) | 45 (0.10) | 24.70 | Ref. | Ref. |
|  | **No. at Risk** | **Mania Diagnosis^A^** | **Mania Diagnosis**  **1 Year** | **Mania Diagnosis**  **3 Years** | **Mania Diagnosis**  **5 Years** | **Crude Rate^B^** | **Age and Sex Overlap Weighted HR (95%CI)^B^** | **Overlap Weighted HR (95%CI)^B,C^** |
| ***Only 1 ED Visit Required for Diagnosis*** | |  |  | **No. (%)** |  |  |  |  |
| **Comparator 1: General Population** | |  |  |  |  |  |  |  |
| Acute Care Visit Involving Hallucinogens | 7,285 | 228 | 86 (1.18) | 152 (2.09) | 188 (2.58) | 771.19 | 25.66 (19.00, 34.66) | 5.88 (3.60, 9.60) |
| General Population | 70,916 | 160 | 24 (0.03) | 59 (0.08) | 101 (0.14) | 29.81 | Ref. | Ref. |

***Note.*** ED = Emergency department. HR = Hazard ratio. CI = Confidence interval.

^A^Diagnosis over maximum follow up period available.

^B^Rate or hazard ratios at 3-years of follow up.

^C^Weighted for age, sex, neighbourhood income quintile, rurality, immigration status, homelessness, past five-year outpatient, ED, and hospital-based care for mental health (anxiety, mood disorder, self-harm, schizophrenia, and other) and substance use (alcohol, stimulants, cannabis, opioids, amphetamines, poly-substance, other).

^D^Supressed to comply with privacy requirements at ICES.

# Table E. Covariate balance before and after overlap weighting, cannabis visit secondary analysis

| **Variable** | **Unweighted Hallucinogen Acute Care** | **Unweighted Cannabis Acute Care** | **Unweighted SMD** | **Weighted Hallucinogen Acute Care** | **Weighted Cannabis Acute Care** | **Weighted SMD** |
| --- | --- | --- | --- | --- | --- | --- |
| **Overall** | 7,285 | 102,931 | . | 65938 | 6593.8 | . |
| **Sex** |  |  |  |  |  |  |
| Female | 28.54% | 37.95% | 0.201 | 29.23% | 29.23% | 0.000 |
| **Age** |  |  |  |  |  |  |
| Mean | 27.39 | 28.81 | 0.121 | 27.43 | 27.43 | 0.000 |
| **Income Quintile** |  |  |  |  |  |  |
| Q1 | 28.83% | 28.93% | 0.002 | 28.71% | 28.71% | 0.000 |
| Q2 | 20.78% | 21.09% | 0.008 | 20.82% | 20.82% | 0.000 |
| Q3 | 17.45% | 17.97% | 0.014 | 17.54% | 17.54% | 0.000 |
| Q4 | 16.32% | 16.32% | 0.000 | 16.40% | 16.40% | 0.000 |
| Q5 | 15.31% | 14.91% | 0.011 | 15.30% | 15.30% | 0.000 |
| Missing | 1.32% | 0.78% | 0.053 | 1.24% | 1.24% | 0.000 |
| **Rurality** |  |  |  |  |  |  |
| Rural | 10.68% | 11.69% | 0.032 | 10.85% | 10.85% | 0.000 |
| Urban | 88.29% | 87.75% | 0.017 | 88.20% | 88.20% | 0.000 |
| Missing | 1.03% | 0.56% | 0.053 | 0.95% | 0.95% | 0.000 |
| **Long Term Resident of Canada** | |  |  |  |  |  |
| Yes | 8.04% | 8.56% | 0.019 | 8.08% | 8.08% | 0.000 |
| **Document History of Homelessness** | |  |  |  |  |  |
| Yes | 11.59% | 6.41% | 0.182 | 10.63% | 10.63% | 0.000 |
| **Substance Use Acute Care Visits in Past 5 Years** | | |  |  |  |  |
| Alcohol | 30.93% | 25.82% | 0.114 | 30.03% | 30.03% | 0.000 |
| Cocaine | 17.34% | 10.22% | 0.208 | 16.23% | 16.23% | 0.000 |
| Amphetamines | 16.35% | 6.12% | 0.328 | 14.62% | 14.62% | 0.000 |
| Opioids | 18.63% | 8.51% | 0.299 | 17.11% | 17.11% | 0.000 |
| Polysubstance | 26.73% | 11.63% | 0.391 | 24.47% | 24.47% | 0.000 |
| Other | 6.89% | 2.89% | 0.186 | 6.04% | 6.04% | 0.000 |
| **Mental Health Acute Care Visits in Past 5 Years** | | |  |  |  |  |
| Mood | 17.83% | 20.83% | 0.076 | 17.77% | 17.77% | 0.000 |
| Anxiety | 25.70% | 25.48% | 0.005 | 25.23% | 25.23% | 0.000 |
| Schizophrenia | 12.60% | 13.58% | 0.029 | 12.37% | 12.37% | 0.000 |
| Self-Harm | 21.67% | 12.39% | 0.249 | 20.23% | 20.23% | 0.000 |
| Other | 9.84% | 8.61% | 0.043 | 9.50% | 9.50% | 0.000 |
| **Outpatient Mental Health and Addiction Visits in Past 5 Years** | | | |  |  |  |
| Family medicine | 73.88% | 70.70% | 0.071 | 73.26% | 73.26% | 0.000 |
| Psychiatry | 43.50% | 41.19% | 0.047 | 42.72% | 42.72% | 0.000 |

***Note.*** SMD = Standardized mean difference.

# Table F. Risk of mania in individuals with acute care involving hallucinogens compared to the general population, sensitivity analysis stratified by age and sex

|  | **No. at Risk** | **Mania Diagnosis^A^** | **Mania Diagnosis  1 Year** | **Mania Diagnosis  3 Years** | **Mania Diagnosis  5 Years** | **Crude Rate^B^** | **Age and Sex Overlap Weighted HR (95%CI)^B^** | **Overlap Weighted HR (95%CI)^B,C^** |
| --- | --- | --- | --- | --- | --- | --- | --- | --- |
| **Comparator 1: General Population** | |  | **No. (%)** | | |  |  |  |
| **Under Age 25** |  |  |  |  |  |  |  |  |
| Acute Care Visit Involving Hallucinogens | 3739 | 86 | 34 (0.91) | 56 (1.50) | 72 (1.93) | 536.75 | 22.00 (13.73, 35.25) | 6.40 (2.87, 14.27) |
| General Population | 36305 | 84 | Suppressed^B^ | 25 (0.07) | 48 (0.13) | 24.31 | Ref. | Ref. |
| **Age 25 or older** |  |  |  |  |  |  |  |  |
| Acute Care Visit Involving Hallucinogens | 3546 | 62 | 30 (0.85) | 48 (1.35) | 54 (1.52) | 513.19 | 30.05 (17.07, 52.90) | 5.40 (2.23, 13.08) |
| General Population | 34611 | 30 | Suppressed^B^ | 16 (0.05) | Suppressed^B^ | 16.82 | Ref. | Ref. |
| **Males** |  |  |  |  |  |  |  |  |
| Acute Care Visit Involving Hallucinogens | 5206 | 102 | 44 (0.85) | 75 (1.44) | 91 (1.75) | 531.10 | 28.66 (18.35, 44.76) | 7.54 (3.55, 16.01) |
| General Population | 50716 | 80 | Suppressed^B^ | 26 (0.05) | 46 (0.09) | 18.38 | Ref. | Ref. |
| **Females** |  |  |  |  |  |  |  |  |
| Acute Care Visit Involving Hallucinogens | 2079 | 46 | 20 (0.96) | 29 (1.39) | 35 (1.68) | 511.93 | 19.09 (10.24, 35.60) | 3.50 (1.28, 9.55) |
| General Population | 20200 | 34 | Suppressed^B^ | 15 (0.07) | 23 (0.11) | 26.57 | Ref. | Ref. |

***Note.*** HR = Hazard ratio. CI = Confidence interval.

^A^Diagnosis over maximum follow up period available

^B^Supressed to comply with privacy requirements at ICES
